# Critical role of formaldehyde during methanol conversion to hydrocarbons

**DOI:** 10.1038/s41467-019-09449-7

**Published:** 2019-04-01

**Authors:** Yue Liu, Felix M. Kirchberger, Sebastian Müller, Moritz Eder, Markus Tonigold, Maricruz Sanchez-Sanchez, Johannes A. Lercher

**Affiliations:** 1 Department of Chemistry and Catalysis Research Center, Technische Universität München, Lichtenbergstr. 4, 85747 Garching, Germany; 2 Clariant Produkte (Deutschland) GmbH, Waldheimer Straße 13, 83052 Bruckmühl, Germany

## Abstract

Formaldehyde is an important intermediate product in the catalytic conversion of methanol to olefins (MTO). Here we show that formaldehyde is present during MTO with an average concentration of ~0.2 C% across the ZSM-5 catalyst bed up to a MeOH conversion of 70%. It condenses with acetic acid or methyl acetate, the carbonylation product of MeOH and DME, into unsaturated carboxylate or carboxylic acid, which decarboxylates into the first olefin. By tracing its reaction pathways of ^13^C-labeled formaldehyde, it is shown that formaldehyde reacts with alkenes via Prins reaction into dienes and finally to aromatics. Because its rate is one order of magnitude higher than that of hydrogen transfer between alkenes on ZSM-5, the Prins reaction is concluded to be the major reaction route from formaldehyde to produce dienes and aromatics. In consequence, formaldehyde increases the yield of ethene by enhancing the contribution of aromatic cycle.

## Abstract (web summary)

The recognition of the importance of HCHO in methanol to olefins (MTO) makes it imperative to quantify its concentration in the reaction and distribution over the catalyst bed. Here the authors quantify the concentration level of HCHO and its distribution along the catalyst bed in MTO, and explores its role in methanol conversion.

## Introduction

The conversion of methanol to hydrocarbons (MTH) is considered as a promising route of converting gas and coal to fuels and chemicals via methanol^1–3^. By adjusting catalysts and reaction conditions, the product distribution shifts from gasoline-range (methanol to gasoline; MTG) to lower olefin-range products (methanol to olefins; MTO)^1^. As a consequence, methanol conversion has been commercialized in different variants^1,4^. The central mechanism consists of two catalytic cycles^5–7^ interconverting surface species (hydrocarbon pool)^8,9^. One is called olefin-cycle and it is dominated by methylation of light alkenes, followed by cracking of the larger alkenes. The other is called aromatic-cycle catalyzing methylation of aromatic molecules followed by cracking of a side chain. The fast propagation of these two cycles is responsible for the autocatalytic nature of the MTH reaction. The relative contribution of each cycle depends on the local concentrations of hydrocarbon species within the zeolite^10^.

A mechanistic description focused solely on hydrocarbons as key compounds may lead, however, to a rather incomplete description of the interlinked processes. As far back as 1984, evidence of formaldehyde formation under the conditions of methanol conversion was given. Kubelková et al.^11^ reported formaldehyde and methane formed by methanol disproportionation on H-ZSM-5 at 670 K and low methanol pressures (1–3 Pa). Hutchings et al. observed methane before C_2+_ hydrocarbons formation at low methanol coverage, supporting these results^12,13^. On the basis of these results a methane-formaldehyde mechanism leading to first C–C bond was proposed by Tajima et al.^14^ In spite of these first reports, formaldehyde in methanol conversion did not attract much attention until recently. Theoretical calculations^15–17^ and dedicated experiments^15,17,18^ showed possible pathways forming the first C–C bond and first olefin from HCHO in a subtle interplay with Brønsted acid and extra-framework Al sites. Experimental observations of strong deactivation in presence of HCHO^19,20^ and of the O-containing surface species, were attributed to reaction products of HCHO, strongly adsorbed on the zeolite acid sites^21–23^. While always being present during MTO conversion at least in very low concentrations, it promotes the formation of non-olefinic byproducts^24,25^ and accelerates deactivation^19–21,26,27^. The recognition of the importance of HCHO in MTO makes it imperative to quantify its concentration in the reaction and distribution over the catalyst bed. However, the very low concentration of HCHO and its high reactivity on acid sites set obstacles in such quantitative studies.

Generation of HCHO under MTO conditions occurs via several pathways, including hydride transfer between two methanol molecules (Rxn 1)^10–13,15^, thermal or reactor-wall catalyzed decomposition of methanol (Rxn 2)^18^ and hydrogen transfer from methanol to alkenes on Lewis acid sites (LAS) (Rxn 3)^24^.

1 $$2{\mathrm{CH}}_3{\mathrm{OH}} \to {\mathrm{HCHO}} + {\mathrm{CH}}_4 + {\mathrm{H}}_2{\mathrm{O}}$$

2 $${\mathrm{CH}}_3{\mathrm{OH}} \to {\mathrm{HCHO}} + {\mathrm{H}}_2$$

3 $${\mathrm{CH}}_3{\mathrm{OH}} + {\mathrm{Alkene}} \to {\mathrm{HCHO}} + {\mathrm{Alkane}}$$

The present study quantifies the concentration level of HCHO and its distribution along the catalyst bed in MTO, and explores the role of formaldehyde as intermediate in two critical stages of methanol conversion. We examine rigorously the participation of formaldehyde in the formation of the first olefinic product on the one hand and its impact on product distribution and deactivation of an H-ZSM-5 catalyst on the other hand. Insight into these elementary steps will help to tailor catalysts to higher alkene selectivity, while extending the useful lifetime of the catalysts.

## Results

### Formaldehyde detection in MTO

**Fig. 1:**
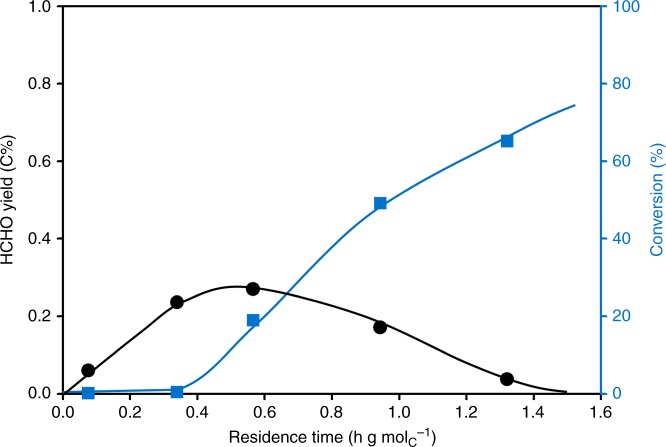
Methanol conversion and the yield of HCHO as a function of residence time. Reaction conditions: DME 90 mbar, H-ZSM5 (Si/Al 90 steamed) 475 °C

Methanol decomposes into HCHO under typical reaction conditions employed in this study (MTO conditions)^18,21–24^. The concentration of the intermediately formed HCHO has not been discussed until now, because the combination of low concentrations and high reactivity makes this very challenging under typical reaction conditions reported. To achieve quantification, we turn to very low conversions. A blank test shows only a conversion of MeOH to 0.01 C% methane and 0.01 C% HCHO, while with H-ZSM-5 a higher conversion was observed. Table 1 shows a typical effluent composition at a methanol (+DME) conversion of only 0.24 C% on H-ZSM-5 at 475 °C. Methane is the dominant product with a yield of 0.12 C%, and HCHO has a yield of 0.06 C%. The rest are CO and CO_2_, with a yield of 0.06 C%. The olefin yield was very low at these conditions, and only a trace concentration of ethene, below 0.01 C%, was detected. The amount of H_2_ was below the detection limit. This shows that MeOH/DME is converted to HCHO with a selectivity as high as 25% before alkenes are formed in appreciable amounts and the hydrocarbon pool has evolved. In Fig. 1 it is shown that by increasing the residence time the yield of HCHO increased to a yield maximum of 0.27 C% at ~20% conversion of MeOH, and then it decreased gradually with higher conversions to levels below the detection limit. These results directly establish the presence of HCHO in H-ZSM-5 under MTO reaction conditions and its concentration evolution with the conversion of MeOH. We investigate next in which steps of the complex reaction network of methanol to olefins does HCHO participate.

**Table 1:** Stream composition in methanol reaction over H-ZSM-5 at a conversion of 0.24%^a^

Effluent molecules	Effluent composition (C%)	Selectivity (C%)
MeOH + DME	99.76	
CH_4_	0.12	50
HCHO	0.06	25
CO + CO_2_	0.06^b^	25
C_2_H_4_	<0.01	<4
H_2_	<0.01 (H%)^c^	—

^a^Conditions: 475 °C, DME 90 mbar, H-ZSM5 (Si/Al 90 steamed), W/F 0.076 h·g_(cat)_·mol_C_^−1^

^b^Estimated based on C balance

^c^Below detection limit

**Fig. 2:**
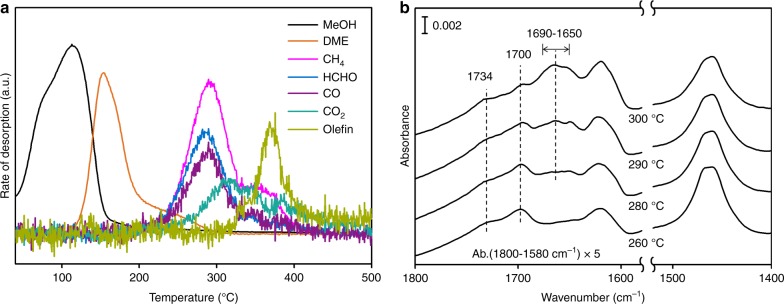
Surface reaction of MeOH adsorbed on H-ZSM-5 with increasing temperature. **a** Desorbed products in gas phase; **b** IR spectrum of corresponding surface species on H-ZSM-5 taken in situ. Reaction conditions: H-ZSM-5 (Si/Al 15) 25 mg saturated under 3 mbar MeOH subsequently outgassed under vacuum, afterwards ramping temperature with 3 °C min^−1^ under vacuum

Having established that HCHO is a main product at low MeOH/DME conversions before alkenes are detected in significant concentrations in the products, we use surface reactions of adsorbed MeOH on H-ZSM-5 to better understand the possible reaction pathways. Figure 2 shows the evolution of gaseous products and surface species from H-ZSM5 saturated with 3 mbar MeOH as a function of temperature. With increasing temperature, MeOH desorption reached a maximum at 120 °C, while DME had maximum at 180 °C with formation extending to 300 °C (Fig. 2a). Decomposition and disproportionation products from MeOH, including CH_4_, HCHO and CO, were detected from 220 to 400 °C with maxima at 290 °C, forming a mixture of C_1_ species. Alkenes appeared at 300 °C and reached a maximum at 380 °C. This agrees with previously reported results, linking the formation of first C–C bond in MTH to the presence of small concentrations of CO^18^. In a recent report, Wu et al. observed a simultaneous appearance of ethene and propene with CH_4_ and HCHO, hence proposing a direct C–C formation from MeOH, DME, surface methoxy or trimethyloxonium ion^28,29^. While we cannot establish the experimental differences, our present study unequivocally identified that olefin appeared after CH_4_, HCHO and CO strongly suggesting that olefin formation follows a different pathway than that Wu et al. proposed. Noticeably, CO_2_ was also observed after MeOH decomposition and before the onset of olefin desorption. The formation of CO_2_ prior to the formation of alkenes in the early stages of the MTH reaction has been attributed to ketonic decarboxylation of two acetic acid molecules into acetone and CO_2_^18^. The present results suggest, however, that this pathway is minor, because acetone was not detected under the present reaction conditions.

IR spectra recorded during this process show the formation and evolution of carbonyl-containing species during the MeOH surface reactions (Fig. 2b). At 260 °C, four bands were observed between 1800 and 1400 cm^−1^: (i) bands of the deformation vibration of water at 1630 cm^−1^, (ii) bands of C–H deformation vibrations at 1460–1470 cm^−1^ (O–CH_3_)^30^, and (iii) two bands of C=O stretching vibrations at 1700 cm^−1^ attributed to acetate (O–CO–CH_3_)^31,32^ and at 1734 cm^−1^ to formate (O–CO-H) groups^33^, respectively. At this temperature, gas phase analysis showed that DME, HCHO, CO and CH_4_ evolved. We hypothesize, therefore, that these C_1_ species are involved in the formation of the surface species observed in the IR spectra.

**Fig. 3:**
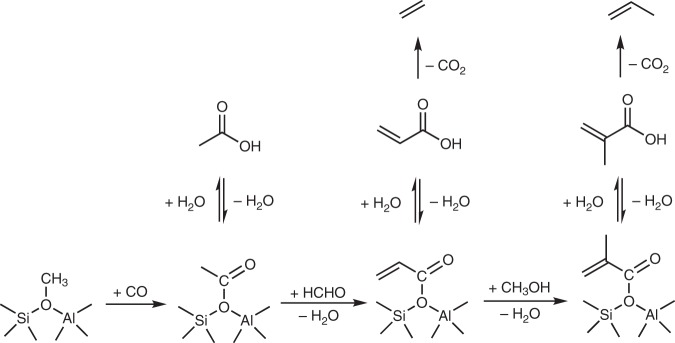
Schematic illustration of the proposed reaction pathways for the formation of alkenes. Surface methoxy group undergoes carbonylation into acetyl group and desorbs as acetic acid, which stepwise converts to unsaturated carboxylic acid, e.g. acrylic acid and methacrylic acid. Ethene and propene are formed via decarboxylation of the unsaturated carboxylic acids

The methoxy group is formed by dissociative adsorption of MeOH/DME on Brønsted acid sites. Acetate groups are formed by CO insertion into the O–CH_3_ bond of methoxy groups^32,34–38^ while formate groups are attributed to be the products of the disproportionation of HCHO under hydrothermal conditions^39^. With reaction progress (here observed when temperature increased from 280 to 300 °C), the acetate C = O stretching vibrations at 1700 cm^−1^ shifts to 1690–1650 cm^−1^. This red shift is attributed to the transformation of acetate groups into unsaturated carboxylates, i.e., acrylate, making conjugated carbonyl groups. This reaction went through the condensation of HCHO at the acetate methyl group (Fig. 3)^40^. The unsaturated carboxylates have also been proposed to convert, via stepwise condensations with HCHO, to O-containing species, strongly interacting with BAS^21^. Note that formation of this unsaturated carboxylates occurred in parallel with the CO_2_ evolution at 280 °C, indicating that partial decarboxylation took place. The evolution of alkenes was then observed at 300 °C (Fig. 2a). This strongly suggests that decarboxylation of unsaturated carboxylic acids plays a role in the formation of the first olefinic products (Fig. 3). An alternative pathway, the methylation of acetate-derived ketene to propionate followed by decarbonylation^36–38^, may also occur in parallel, but is less important under the applied condition here, because neither ketene nor propionate were observed.

**Fig. 4:**
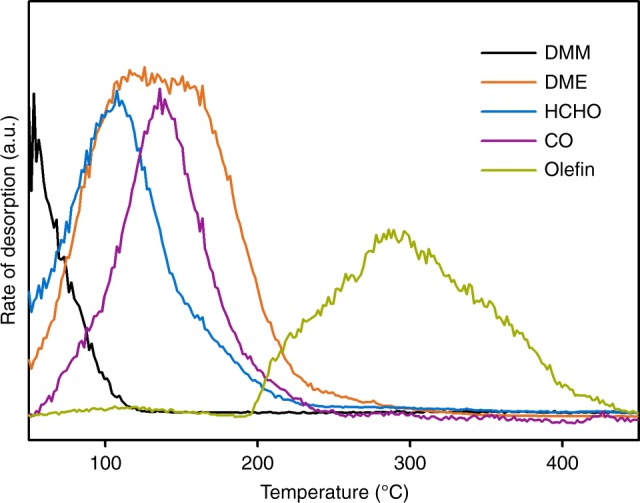
Surface reaction of DMM adsorbed on H-ZSM-5 with increasing temperature. Reaction conditions: H-ZSM-5 (Si/Al 15) 25 mg saturated under 1 mbar DMM, subsequently outgassed under vacuum, afterwards ramping temperature with 3 °C min^−1^ under vacuum

A similar temperature-programmed surface reaction was performed with dimethoxymethane (DMM) instead of MeOH (Fig. 4). On H-ZSM-5, DMM decomposes into equimolar concentration of HCHO and DME below 100 °C. Thus, the surface reaction of DMM at T > 100 °C represents the reaction of a mixture of HCHO and DME on H-ZSM-5. The evolution of alkenes started in this case at ~200 °C, while in pure MeOH alkenes did not appear until 300 °C (Fig. 2a). Converting MeOH required temperatures above 200 °C to generate HCHO and CO. In presence of HCHO and CO the reaction started already below 200 °C, facilitating the initiation of the hydrocarbon pool at low temperatures.

### Participation of formaldehyde in the dual-cycle mechanism

Having shown how HCHO participates in the formation of the first olefin, we investigate next its participation in the dual-cycle mechanism. Because HCHO is H-poor, incorporation into products must increase the selectivity to aromatic molecules^20,24^, and in turn the selectivity to ethene, formed in the aromatic cycle^20^. As the formation of aromatic molecules has been associated to deactivation of the zeolite catalysts, we hypothesize that the higher concentration of HCHO in the reacting mixture leads to faster deactivation of the catalyst^20^.

In order to show the most relevant conversion pathways of HCHO, ^13^C-labeled HCHO was co-fed with MeOH. Table 2 shows the selectivity to hydrocarbon products when feeding pure MeOH and MeOH with 5 C% HCHO at comparable conversion levels (88.8 C% and 75.8 C%, respectively). For pure MeOH feed, propene and butene were the major products, with selectivities of 36.9 C% and 20.3 C%, respectively. Ethene selectivity was only 3.0 C%, in good agreement with the low yield of aromatics (2.4 C%). The products indicate that under the selected reaction conditions the aromatic cycle was less important than the olefin cycle. The selectivity to C_1-4_ alkanes was at the same low level as aromatics, indicating low rate of hydrogen transfer reactions.

**Table 2:** Conversion and product selectivity in MTO reaction with and without H^13^CHO^a^

Feed composition	MeOH	MeOH + 5 C% H^13^CHO
Conversion (C%)	88.8	75.8
Product selectivity (C%)		
Ethene	3.0	8.6
Propene	36.9	28.1
Butene	20.3	15.8
Dienes^b^	0.4	0.7
Aromatics	2.4	12.2
C_1-4_ alkanes	3.1	2.8
C_5+_ aliphatics	20.2	20.1

^a^Reaction conditions: H-ZSM-5 (Si/Al 90 steamed), W/F 1.82 h·g_cat_·mol_(MeOH+HCHO)_^−1^, MeOH 180 mbar, H_2_O 60 mbar, or MeOH 171 mbar, H^13^CHO 9 mbar, H_2_O 60 mbar, 475 °C

^b^Butadiene and pentadiene

When HCHO was co-fed with MeOH, the selectivity to H-poor products, i.e., dienes and aromatics, increased drastically. The selectivity to aromatic molecules increased five-fold from 2.4 to 12.2 C%. The ethene selectivity increased from 3.0 to 8.6 C%. In parallel, the selectivities to propene and butene decreased from 36.9 C% to 28.1 and from 20.3 to 15.8 C%, respectively. These changes indicate that in presence of HCHO the olefin cycle decreased in importance. The selectivity to C_1-4_ alkanes did not change, which indicates that the hydrogen transfer rate was not affected by the presence of HCHO. Thus, the increase of dienes and aromatics is concluded to be the result of a direct reaction between alkenes and HCHO.

**Fig. 5:**
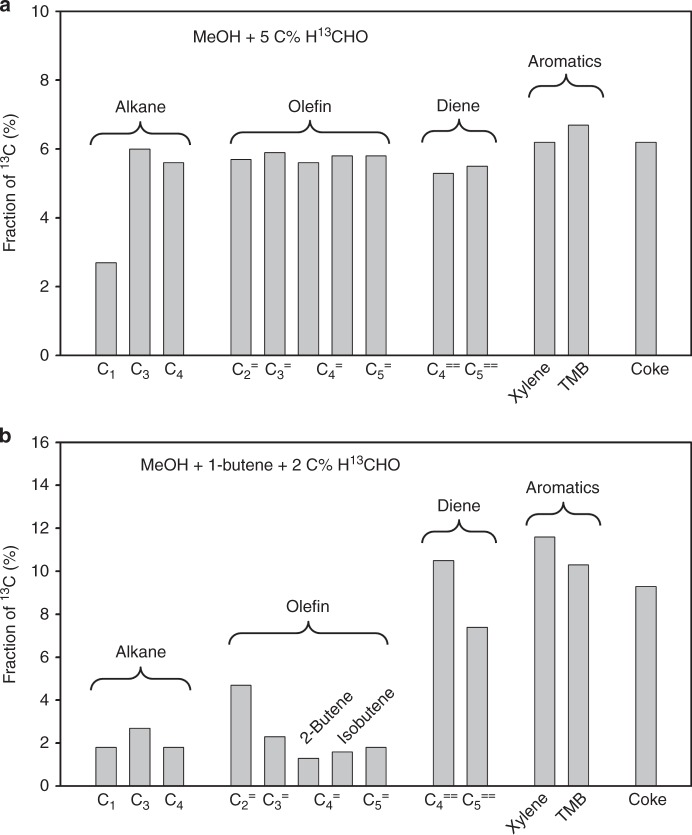
Fraction of ^13^C in hydrocarbon products in the reaction of MeOH cofed with H^13^CHO. **a** MeOH cofed with 5 C% H^13^CHO, MeOH conversion 75%. **b** MeOH cofed with 1-butene and 2 C% H^13^CHO; MeOH conversion 100%, butene decreased from 57% in the feedstock to 24% in the gas products. C_2_^=^, C_3_^=^, C_4_^=^ and C_5_^=^ refer to ethene, propene, butene and pentene, respectively; C_4_^==^ and C_5_^==^ refer to butadiene and pentadiene respectively. Reaction conditions: **a** W/F 1.82 h·g_(cat)·_mol_(MeOH+HCHO)_^−1^, MeOH 171 mbar, H^13^CHO 9 mbar, H_2_O 60 mbar, 475 °C; **b** W/F 1.30 h·g_(cat)·_mol_(MeOH+HCHO)_^−1^, MeOH 171 mbar, H^13^CHO 9 mbar, H_2_O 60 mbar, 1-butene 60 mbar, 475 °C. See Supplementary Methods 1 and 2 for the determination of ^13^C fraction

The distribution of ^13^C in the products can be used to deduce the reaction pathways in which HCHO is preferentially incorporated into hydrocarbons. Figure 5a shows the fraction of each hydrocarbon product containing ^13^C. All hydrocarbon products had a similar percentage of ^13^C incorporated, within 5 to 6%, corresponding to the total ^13^C content of the feed. Only methane showed a significantly lower fraction of 2.7%. This uniform distribution of ^13^C in the product mixture and particularly the value close to ^13^C fraction in the feed (accounting for the natural abundance of 1% ^13^C in MeOH) indicates a fast scrambling of ^13^C during reaction.

The scrambling is hypothesized to result from the fast interconversion of MeOH with H^13^CHO via hydride transfer from MeOH to a protonated H^13^CHO on a BAS, which generates a ^13^C-labeled MeOH (^13^CH_3_OH) and an unlabeled HCHO (Rxn 4).

4 $${\mathrm{CH}}_3{\mathrm{OH}} + {\mathrm{H}}^{13}{\mathrm{CHO}} \to {\mathrm{HCHO}} + \,^{13}{\mathrm{CH}}_3{\mathrm{OH}}$$

This hypothesis is supported by the detection of 5.5% of ^13^C labelled MeOH and concurrently HCHO with only 8.7% ^13^C at MeOH conversions as low as 5 C%. The low ^13^C fraction in methane indicates that it is formed mainly in reactions during the initiation stage of the methanol conversion to hydrocarbons (Rxn 1), occurring before and in parallel to the MeOH/HCHO scrambling in Rxn 4. Thus, the fast scrambling of MeOH with H^13^CHO before the appearance of alkenes does not allow tracking the conversion pathway of H^13^CHO.

It has been reported that co-feeding alkenes, such as propene and butene quickly initiates the olefin cycle and subsequently also the aromatic cycle^4^. Although under such conditions the hydrogen transfer from MeOH to H^13^CHO still exist, the extent of scrambling is hypothesized to be significantly reduced, because of the accelerated rate of MeOH (or HCHO) consumption in forming C–C bonds by alkylation. Therefore, 1-butene was co-fed with MeOH and H^13^CHO (Fig. 5b). A higher incorporation of ^13^C was observed in dienes and aromatics: 10.5% in butadiene, 7.4% in pentadiene, 11.6% in xylene and 10.3% in trimethylbenzene (TMB). In contrast, alkanes had only about 2% of ^13^C. Within alkenes, ethene had the highest ^13^C fraction (4.7%); for propene it was 2.3% and for butene and pentene even lower (1.3% for 2-butene, 1.6% for isobutene and 1.8% for pentene). The total ^13^C content in the gas products was 2.9%, very close to the 3.1% ^13^C in the feedstock (2% from H^13^CHO and 1.1% from natural abundance in MeOH and butene), in which the 0.2% difference could be those incorporated in ^13^CO, ^13^CO_2_ or coke. These results show that HCHO participates in both cycles as a C_1_ source. Ethene is formed in the aromatic cycle and the high incorporation of ^13^C in ethene and aromatic molecules indicates a high involvement of H^13^CHO in the aromatic cycle. Both pentene and isobutene are products and intermediates in olefin cycle. Although the direct skeletal isomerization of the cofed 1-butene to isobutene is possible, this pathway has only a minor contribution on H-ZSM-5 and most isobutene is generated from cracking of higher olefins^41,42^. Therefore, their low incorporation of ^13^C indicates a minor participation of H^13^CHO in the olefin cycle.

Isobutene is chosen as indicator of the olefin cycle, because the other two butene isomers are either the co-fed reactant (1-butene) or can be formed by 1-butene isomerization on BAS without passing the olefin cycle (2-butene). Propene is generated in both the aromatic and the olefin cycle^1,2,4^, showing in consequence a ^13^C incorporation level intermediate between ethene and isobutene. The preferred ^13^C enrichment of dienes and aromatics supports earlier conclusions that HCHO leads to H-poor products at a rate that is higher than that of hydrogen transfer between hydrogen poor and hydrogen rich hydrocarbon intermediates.

5 $${\mathrm{C}}_4{\mathrm{H}}_8 + {\mathrm{C}}_4{\mathrm{H}}_8 \to {\mathrm{C}}_4{\mathrm{H}}_6 + {\mathrm{C}}_4{\mathrm{H}}_{10}$$

6 $${\mathrm{C}}_4{\mathrm{H}}_8 + {\mathrm{HCHO}} \to {\mathrm{C}}_5{\mathrm{H}}_8 + {\mathrm{H}}_2{\mathrm{O}}$$

An alkene, for e.g., butene, can react into a diene in MTO via two pathways, hydrogen transfer with another alkene (Rxn 5) and Prins reaction with a formaldehyde (Rxn 6). Unlike hydrogen transfer, the Prins reaction has not attracted much attention until recently. Earlier reports have, however, noted the possibility of Prins type reaction for the formation of dienes and aromatics without experimental evidence^20,24^. Comparing the isotope distribution allows now unequivocally establishing the importance of the two routes. If hydrogen transfer were the dominant path of diene formation (Rxn 5), butadiene and pentadiene would have a ^13^C labelling similar to that of butene and pentene, respectively. The fact that eight times more ^13^C was found in butadiene (10.5%) than in n-butene (1.3%) and over four times more ^13^C in pentadiene (7.4%) than in pentene (1.8%) when MeOH was reacted together with 1-butene and 2 C% H^13^CHO, allowed us to rule out hydrogen transfer as the main pathway to dienes. Moreover, the rate of hydrogen transfer has been reported to increase by one order of magnitude by the simultaneous presence of MeOH and alkenes, attributed to the reaction pathway involving hydrogen transfer from MeOH to an alkene^24^. Such reaction generates formaldehyde in situ, which, as discussed above, reacts subsequently by Prins reaction converting a second alkene to a diene (Rxn 7). Therefore, we conclude that the Prins reaction is the dominant pathway for diene formation.7

This conclusion is further supported by an additional experiment in which 1-butene was reacted with H^13^CHO in absence of MeOH. The resulting pentadiene from this reaction had a labelling of ~20% ^13^C (Supplementary Fig. 1), indicating an incorporation of one ^13^C in each pentadiene molecule via a Prins type reaction (Rxn 8). In the reaction of MeOH with butene and H^13^CHO, the incorporation of ^13^C in pentadiene was much lower (7.4% ^13^C, Fig. 5b). We speculate that this is caused by H^13^CHO being partially interconverted with unlabeled HCHO generated in situ from MeOH via Rxn 4 and Rxn 7.

8 $${\mathrm{C}}_4{\mathrm{H}}_8 + {\mathrm{H}}^{13}{\mathrm{CHO}} \to \,^{13}{\mathrm{C}}_1{\mathrm{C}}_4{\mathrm{H}}_8 + {\mathrm{H}}_2{\mathrm{O}}$$

**Fig. 6:**
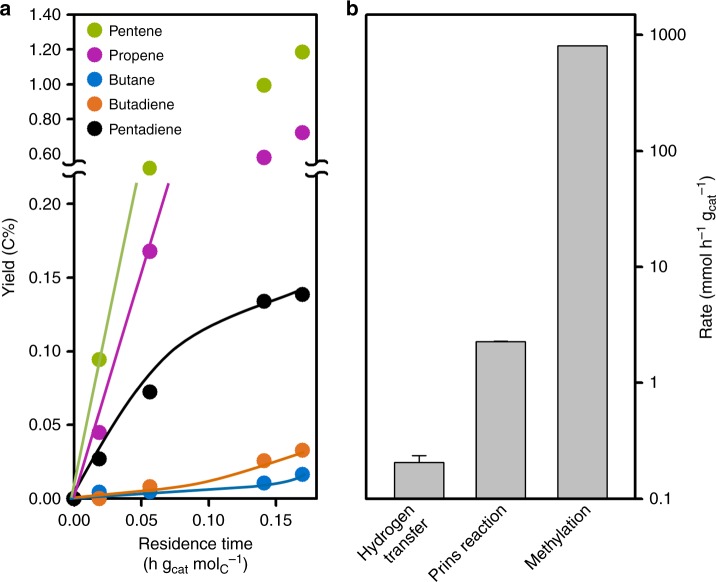
Reaction of 1-butene with HCHO over H-ZSM-5. **a** Product yield as a function of residence time. Reaction condition: 1-butene 45 mbar, HCHO 0.32 mbar, H_2_O 22.5 mbar, 475 °C. **b** Reaction rates obtained for hydrogen transfer, Prins reaction (initial rates of formation of butane and pentadiene respectively under reaction conditions shown in **a** and methylation (represented by the DME/MeOH conversion rate at ~40% conversion shown in Fig. 1) on H-ZSM-5. Error bar represents the standard error of the reactions rates

After showing the participation of HCHO in the dual cycle via Prins reaction, we discuss the importance of this reaction pathway in typical MTO reaction for the non-olefinic byproduct formation. In order to do so, we compare the reaction rates of Prins reaction and hydrogen transfer between alkenes in H-ZSM-5. To avoid the interference of products directly formed via MeOH routes, we examine these reactions by studying the reaction of 1-butene –chosen as representative of the olefin pool – with HCHO on H-ZSM-5. Figure 6a shows the product yield in the reaction of 45 mbar 1-butene with 0.32 mbar HCHO. The HCHO concentration was chosen as 0.18 C% in the total feed, corresponding to the average concentration derived from the yield of HCHO during MTO reaction at different contact times (as shown in Fig. 1). Butene dimerization and cracking were the dominant reactions leading to a 0.72 C% yield of propene, 1.2 C% yield of pentene and 0.17 C% yield of higher aliphatic products at 0.17 h g_cat_ mol_C_^−1^ residence time (Fig. 6a). In addition, small concentrations of pentadiene, butadiene and butane were formed (Fig. 6a). Pentadiene is the product from Prins reaction of butene with HCHO (Rxn 6) while butane is formed via hydrogen transfer reaction (Rxn 5). Butadiene can be formed both from Prins reaction of propene with HCHO and from hydrogen transfer reaction.

Therefore, the rates of pentadiene and butane formation represent the rates of Prins reaction and hydrogen transfer, respectively. As it can be seen in Fig. 6b, the rate of Prins reaction is one order of magnitude higher than that of hydrogen transfer, even though the concentration of HCHO was two orders of magnitude lower than that of butene. These results provide unequivocal evidence for previous speculations that the Prins reaction is the major route of HCHO being converted to H-poor products in the MTO process, i.e., dienes and aromatics^18,20^. As a reference, the rate of methylation, which represents the rate of the dual cycles, derived from a standard MTO feed (Fig. 1) is also included in Fig. 6b. It can be concluded that the reactions in the dual cycle are dominant in MTO, because the methylation rate is two orders of magnitude higher than the rate of Prins reaction. However, formaldehyde forms aromatics and H-poor products selectively, even if present only in low concentrations. Thus, it impacts the product distribution of the overall MTO process. The presence of HCHO acts in analogy to the established effect of co-feeding small concentrations of aromatics with MeOH on H-ZSM-5^4^, which leads to enhancement of the aromatic cycle, shifting the selectivity of the process towards aromatics and ethene.

### Role of formaldehyde in deactivation

**Fig. 7:**
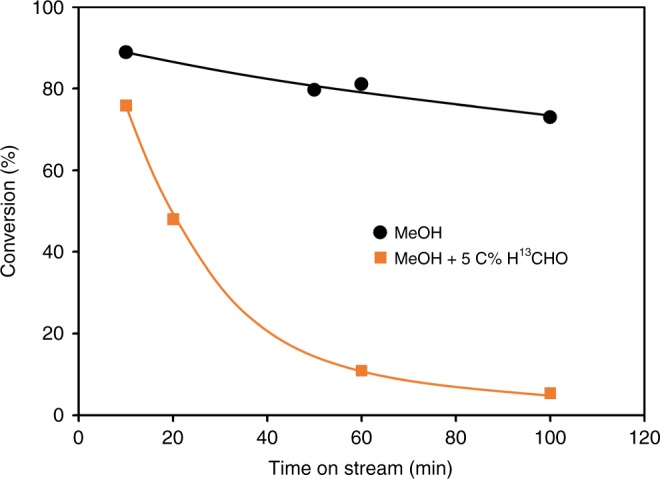
Evolution of MeOH conversion during MTO reaction with time on stream. The reactions in presence and absence of cofed H^13^CHO were compared. Reaction conditions: H-ZSM-5 (Si/Al 90 steamed), W/F 1.82 h·g_cat_·mol_(MeOH+HCHO)_^−1^, MeOH 180 mbar, H_2_O 60 mbar, or MeOH 171 mbar, H^13^CHO 9 mbar, H_2_O 60 mbar, 475 °C

Aromatic molecules are coke precursors in MTO^21,22,25^. The higher yield of aromatics induced by the presence of HCHO will, thus, cause a higher coking and deactivation rates. This is supported by the sharp decline of conversion with time on stream for the reactions of MeOH with 5 C% cofed H^13^CHO in contrast to pure MeOH feeds (Fig. 7). It is shown in Section 2.1 that the presence of HCHO would promote reactivity by facilitating the first olefin formation. However, because of the strong deactivation induced when 5 C% of MeOH is replaced by H^13^CHO, under the same reaction conditions, the conversion dropped below 80% after only 10 min time on stream and to approximately 5% after 100 min. Conversely, when butene was co-fed with MeOH and HCHO, the fast consumption of MeOH and HCHO via alkylation and Prins reaction with butene lead to their full conversion at the contact time studied. The conversion only dropped slightly to 98.5% after 100 min time on stream (Supplementary Fig. 2). This agrees well with previous conclusions that the presence of alkenes drastically prolongs catalyst lifetime^10,21^.

The carbon deposits on H-ZSM-5 using different feeds were analyzed after 100 min time on stream and results are compiled in Table 3. The reaction of pure MeOH feed for 100 min accumulated 1.0 wt.% of coke on catalyst. In contrast, co-feeding 5 C% H^13^CHO increased the deposited coke to 5.2 wt.%. Normalizing the coke concentration to the converted MeOH showed that only 0.084 C% of pure MeOH feed are converted to coke, but 1.3 C% for MeOH co-fed with 5 C% H^13^CHO. We conclude that the high rate of coke formation in presence of HCHO is attributed to the observed higher yield towards H-poor products.

**Table 3:** Coke concentration and extent of ^13^C labelling after 100 min time on stream

Reactants	Coke concentration on catalyst (wt.%)	Coke amount per total converted MeOH (C%)	^13^C fraction in coke (%)
MeOH^a^	1.0	0.084	1.1^b^
MeOH + 5 C% H^13^CHO^a^	5.2	1.3	6.2
MeOH + 1-Butene + 2 C% H^13^CHO^c^	7.7	0.37	10.0

^a^W/F 1.82 h·g_(cat)·_mol_(MeOH+HCHO)_^−1^, MeOH 171 mbar, H^13^CHO 9 mbar, H_2_O 60 mbar, 475 °C

^b^Natural abundance of ^13^C

^c^W/F 1.30 h·g_(cat)·_mol_(MeOH+HCHO)_^−1^, MeOH 171 mbar, H^13^CHO 9 mbar, H_2_O 60 mbar, 1-butene 60 mbar, 475 °C

When butene was co-fed to MeOH and HCHO, 7.7 wt.% coke was deposited, corresponding to 0.37 C% of the total converted MeOH. This lower coke formation per converted MeOH in the presence of butene, is attributed to the successful competition of methylation of butene, decreasing the local concentration of MeOH along the catalyst bed and, as a consequence, the concentration of HCHO (formed by MeOH hydrogen transfer).

The ^13^C content of coke was analyzed by measuring the fraction of ^13^CO and ^13^CO_2_ in total CO and CO_2_ during its combustion in temperature-programmed oxidation. The fast scrambling of ^13^C in H^13^CHO with MeOH (Rxn 4) under MTO conditions causes an almost equal distribution of ^13^C (5–6%) in all products, including coke (6.2% ^13^C) in the reaction of MeOH with 5 C% H^13^CHO. When the ^13^C content of coke was analyzed after co-feeding butene with MeOH and 2 C% H^13^CHO, coke contained 10% ^13^C, which is comparable to the ^13^C percentage found in aromatics (11.6% ^13^C for xylene and 10.3% ^13^C for TMB). This amount of ^13^C in coke corresponds to 0.72 C% of total converted H^13^CHO, which is two-fold higher than the percentage of converted MeOH that ended up in coke (0.37%), showing that HCHO has a higher fraction incorporated than MeOH.

## Discussion

The present experiments show unequivocally that formaldehyde, methane and CO are generated from MeOH under MTO conditions in H-ZSM-5. We have been able to identify key reaction intermediates in the mechanism of formation of alkenes from a C_1_ reacting mixture containing MeOH, CO and HCHO. MeOH and DME react with CO into methyl acetate and acetic acid as the first species containing a C–C bond^18,35–38^. Formaldehyde condenses with surface methyl acetate and acetic acid to form unsaturated carboxylic acids, which then are converted into the first olefin species via decarboxylation. Once the concentration of these olefins in the catalyst surpasses a threshold value, the fast methylation activity of Brønsted acid sites allows for the full development of the MTO dual-cycle reaction network.

Formaldehyde reacts with olefins into dienes via Prins reaction. The Prins reaction is one order of magnitude faster than the hydrogen transfer between two alkenes, which makes it the dominant reaction towards H-poor byproducts, i.e., dienes, aromatics and coke. Even in small concentrations, the presence of HCHO increases the selectivity to aromatics, enhancing the importance of the aromatic cycle in the dual cycle and in turn shifting the process towards a higher selectivity to ethene at expenses of the selectivity to propene and butenes. As an additional consequence, the high yield of aromatics induced by HCHO leads to a high rate of coke formation and to a high rate of deactivation.

Strategies to extend catalyst lifetime should aim, therefore, to minimize the HCHO concentration during the MTO reactions. This could be conceptually achieved by inhibiting its formation or by its fast decomposition. Indeed, many of the improvements in catalyst lifetime reported in the literature can be attributed to reaction conditions in which the chemical potential of MeOH – and thus of HCHO – is reduced in the reactor (via dilution of MeOH^20,26^, co-feeding alkenes^10^, back-mixing products^10,21^ or replacing MeOH by DME^19^).

## Methods

### Catalysts

H-ZSM-5 catalyst with Si/Al 90 was synthesized according to the procedure described by Ong et al.^43^. In brief, Na-ZSM-5 was first synthesized by mixing colloidal silica, Al(NO_3_)_3_‧9H_2_O, NaOH and tetrapropylammonium bromide (TPABr) with a composition of 100 SiO_2_: 0.2 Al_2_O_3_: 5 Na_2_O: 10 TPABr: 4000 H_2_O. After aging, the obtained gel was transferred to an autoclave and kept at 180 °C for 48 h. Then the solid was separated by filtration and washed until pH 8. Afterwards, the powder was dried at 100 °C overnight and calcined with the following sequential steps: (1) rising with 1 °C min^−1^ to 200 °C in flowing He and kept for 3 h; (2) rising with 1 °C min^−1^ to 520 °C in flowing air and kept for 3 h. The obtained Na-ZSM-5 was then transformed into H-ZSM-5 via ion-exchange with NH_4_NO_3_ solution and calcination in flowing air at 520 °C for 3 h. It has a Si/Al ratio of 90 according to atomic absorption spectroscopic analysis. For some experiments, the H-ZSM-5 was steamed at 753 K for 24 h at water vapor pressure of 1 bar prior to usage. Accordingly, the samples are denoted as H-ZSM-5 (Si/Al 90) and H-ZSM-5 (Si/Al 90, steamed). For the TPSR/IR spectroscopy experiment, an H-ZSM-5 with Si/Al 15 was used (named as H-ZSM-5 (Si/Al 15)) which was purchased from Zeolyst. Methanol (≥99.9%) and dimethoxymethane (99%) were supplied by Sigma-Aldrich. ^13^C-labeled HCHO (99 atom % ^13^C) was purchased from Sigma-Aldrich as aqueous solution (20 wt.%).

### TPSR and IR spectroscopy

Temperature-programmed surface reactions (TPSR) of MeOH and DMM were performed in a home-made IR-cell connected to a mass spectrometer. A self-supporting wafer of 25 mg H-ZSM-5 (Si/Al 15) was loaded in the cell center and perpendicular to the IR beam. The H-ZSM-5 (Si/Al 15) has a high acid site concentration and high adsorption capacity of MeOH and DMM, and led, thus, to higher intensities of the bands in the IR spectra. The wafer was first activated at 723 K in vacuum for 1 h. After cooling down to 40 °C, 3 mbar MeOH or 1 mbar DMM was introduced into the cell and kept for 15 min followed by desorption for 30 min under vacuum. Then, the wafer temperature was increased to 500 °C with a rate of 3 °C min^−1^. Desorbed molecules were detected on line using mass spectrometry: m/e 31 for MeOH, m/e 75 for DMM, m/e 45 for DME (after subtracting fragment ion signal of m/e 45 from DMM), m/e 16 for methane, m/e 30 for HCHO (after subtracting fragment ion signal of m/e 30 from MeOH), m/e 44 for CO_2_, m/e 28 for CO (after subtracting fragment ion signal of m/e 28 from CO_2_), m/e 27 for olefins. In-situ IR spectra of the wafer were collected on a Bruker Vertex 70 FTIR spectrometer.

### Temperature-programmed oxidation of coke on spent catalysts

Thermogravimetric analysis (TGA) on a SETARAM Sensys Evo TGA-DSC was utilized to analyze coke deposited on deactivated catalysts. Typically, 10–20 mg of powdered sample was loaded and treated at 200 °C in 16 mL min^−1^ He flow until weight stabilization. Afterwards, the temperature was raised to 650 °C at 5 °C min^−1^ in 16 mL min^−1^ 10 v% O_2_ in He flow and kept for 1 h. The coke amount was obtained from the loss of weight and the formed H_2_O, CO and CO_2_ were detected online with an MS.

### Catalytic testing

Catalytic measurements were performed in a fixed bed quartz reactor with an internal diameter of 6 mm at 475 °C and ambient pressure. The H-ZSM-5 catalysts (200–280 μm) were homogeneously diluted with silicon carbide (ESK-SiC) in the range of 355–500 μm to ensure temperature uniformity. Catalysts were activated at 475 °C for 1 h under He atmosphere before reaction. Methanol and water were introduced into the reactor by an HPLC-pump combine with a direct evaporator. For cofeeding experiments, 1-butene was fed by MFC (Bronkhorst) and ^13^C-formaldehyde solution (20 wt%) was introduced into reactor by mixing with MeOH and the pump-evaporator combination. Via adjusting mixing ratio and liquid flow as well as the butene and He gas flow, the feeding ratio and partial pressures of 1-butene and ^13^C-formaldehyde were varied, and the partial pressure of water was kept constant at 60 mbar. Products were analyzed online on a gas chromatograph (HP 5890) equipped with a HP-PLOTQ capillary column and an FID detector. A mass spectrometer is used to analyze H_2_. Formaldehyde is detected by solving the reaction effluent in water at 2 °C with subsequent stoichiometric Hantzsch reaction as described by Nash^44^ and quantification by a Varian Cary 50 UV-Vis Spectrophotometer. The product yield and selectivity were given on a carbon basis and DME was treated as unconverted Methanol. For the quantification of ^13^C fraction in the products, a certain volume of product stream was collected and analyzed on a GC-MS (Agilent Technologies 7890 B GC, column: Agilent HP-PLOT Q, 30 m, 0.32 mm, 20.00 µm). The analysis of ^13^C incorporation is described in Supporting Information Methods part.

## Supplementary information

Supplementary Information

Source Data

## Data Availability

The source data underlying for Figs. 1, 2, 4–7 are provided as a Source Data file. All other data supporting the findings in this study are available from the authors on request.
