# Supplementary material for: Critical role of formaldehyde during methanol conversion to hydrocarbons
Source: Nat Commun. 2019 Apr 1;10:1462. doi: 10.1038/s41467-019-09449-7 (PMC6443648; doi:10.1038/s41467-019-09449-7)
Supplement: Supplementary file 1 — Supplementary Information [file 41467_2019_9449_MOESM1_ESM.pdf]

Critical role of formaldehyde during methanol conversion to hydrocarbons

Yue Liu[1,‡], Felix M. Kirchberger[1,‡], Sebastian Müller[1], Moritz Eder[1], Markus Tonigold[2], Maricruz Sanchez-Sanchez[1,*] and Johannes A. Lercher[1,*]

[1]Department of Chemistry and Catalysis Research Center, Technische Universität München, Lichtenbergstr. 4, 85747 Garching, Germany

[2]Clariant Produkte (Deutschland) GmbH, Waldheimer Straße 13, 83052 Bruckmühl, Germany

** Corresponding authors: m.sanchez@tum.de, johannes.lercher@ch.tum.de*

‡ *These authors contributed equally to this work*

Supplementary Information

Supplementary Method 1. Analysis of ^{13}C incorporation in gas products.

The degree of ^{13}C incorporation in gas products in MTO was determined by analyzing the MS spectra of each gas product in GC-MS. The normal procedure of analyzing the shift of the molecular ion towards m/e +1 and +2 is hardly applicable in this work because the ^{13}C incorporation is very low and the molecular ions of a few products are less abundant than fragment ions. Therefore, we used a different approach as follow:

(1) For a certain product, we take its MS spectra and find all the fragment ions that have the same number of carbons as the molecular ion in the MS spectra.

(2) Define and calculate "mean weight (m/e) of fragment ions" as intensity weighted fragment ion weight:

$$W_{\text{frag}} = \frac{\sum_{j=m}^{n} j \cdot I_{\text{j}}}{\sum_{j=m}^{n} I_{\text{j}}} \qquad \text{(Eq. 1)}$$

The m and n are the lowest m/e and highest m/e in the fragment ions mentioned in (1); I_{j} is the MS peak intensity of the fragment ion with m/e of j.

(3) The unlabeled product has the mean weight of fragment ions, $W_{\text{frag,unlabel}}$; the ^{13}C incorporated sample has $W_{\text{frag,samp}}$. Then the fraction of ^{13}C in the product, $X_{13\text{C}}$, is calculated by:

$$X_{13\text{C}} = \frac{W_{\text{frag,samp}} - W_{\text{frag,unlabel}}}{N} + 1.1\% \qquad \text{(Eq. 2)}$$

in which N is the number of carbon atoms in the product molecule; 1.1% is the natural abundance of ^{13}C.

<u>The derivation of equation (2):</u>

The product sample is a mixture of unlabeled and ^{13}C labeled molecules. It contains x_0 mole of unlabeled molecule, x_1 mole of one ^{13}C labeled molecule, x_{j} mole of j (number) ^{13}C labeled molecule, till x_{N} mole of N ^{13}C labeled molecule. N is the number of carbon atoms in the product molecule. The unlabeled molecule has the mean weight of fragment ions, $W_{\text{frag,unlabel}}$. The one ^{13}C labeled molecule has the mean weight of fragment ions, $W_{\text{frag,unlabel}} + 1$. The j (number) ^{13}C labeled molecule has the mean weight of fragment ions, $W_{\text{frag,unlabel}} + j$. Then the product sample has a mean weight of fragment ions of $W_{\text{frag,samp}}$.

$$W_{\text{frag,samp}} = \frac{\sum_{j=0}^{N} x_{\text{j}} \left(W_{\text{frag,unlabel}} + j \right)}{\sum_{j=0}^{N} x_{\text{j}}} = W_{\text{frag,unlabel}} + \frac{\sum_{j=0}^{N} x_{\text{j}} j}{\sum_{j=0}^{N} x_{\text{j}}} \qquad \text{(Eq. 3)}$$

The second term on the right in equation (3) is virtually the number of more ^{13}C atoms per product molecule compared to that in unlabeled molecule. Since each product molecule contains N number of carbon atoms, the fraction of ^{13}C in the product, $X_{13\text{C}}$, is thus given by equation 4. The natural abundance (1.1% ^{13}C) of unlabeled molecule is accounted.

$$X_{13\text{C}} = \frac{\sum_{j=0}^{N} x_{\text{j}} j}{\sum_{j=0}^{N} x_{\text{j}}} \cdot \frac{1}{N} + 1.1\% = \frac{W_{\text{frag,samp}} - W_{\text{frag,unlabel}}}{N} + 1.1\% \qquad \text{(Eq. 4)}$$

Supplementary Method 2. Analysis of ^{13}C incorporation in cokes.

The quantification of ^{13}C fraction in cokes was carried by analyzing the generated CO_2 on a mass spectrometer in the temperature programmed oxidation (TPO) of cokes. With the obtained spectra, the intensity ratio of $^{13}CO_2$ (m/e 45) to the sum of the intensity of $^{12}CO_2$ (m/e 44) and $^{13}CO_2$ (m/e 45) was used as the ^{13}C fraction in coke.

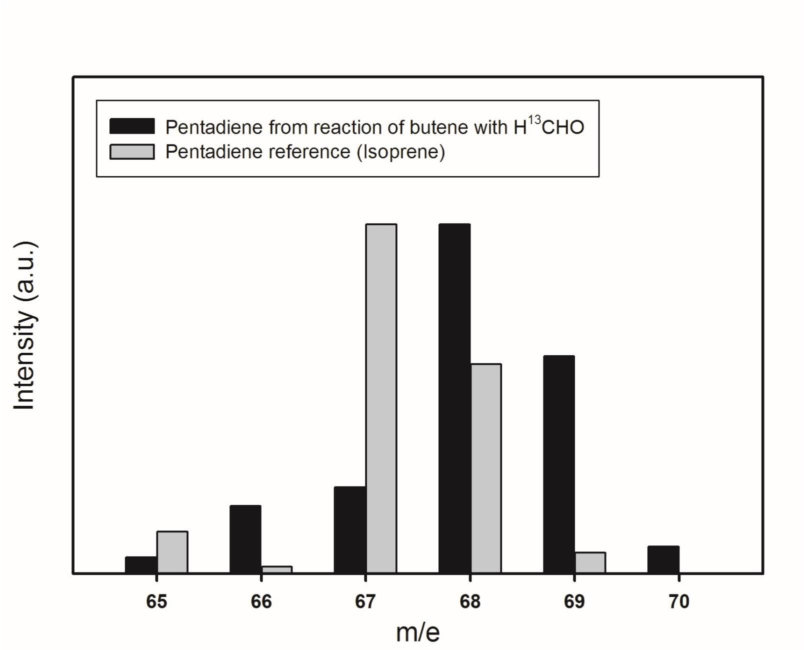

Supplementary Figure 1 | MS of pentadiene from the reaction of butene with $H^{13}CHO$. Reaction conditions: $H^{13}CHO$ 3.8 mbar, butene 1.5 mbar ($H^{13}CHO$/Butene 2.5/1), 35 mL/min N_2 flow, H-ZSM-5 (Si/Al 90 steamed) 35 mg, 475 °C, butene conversion 26%.

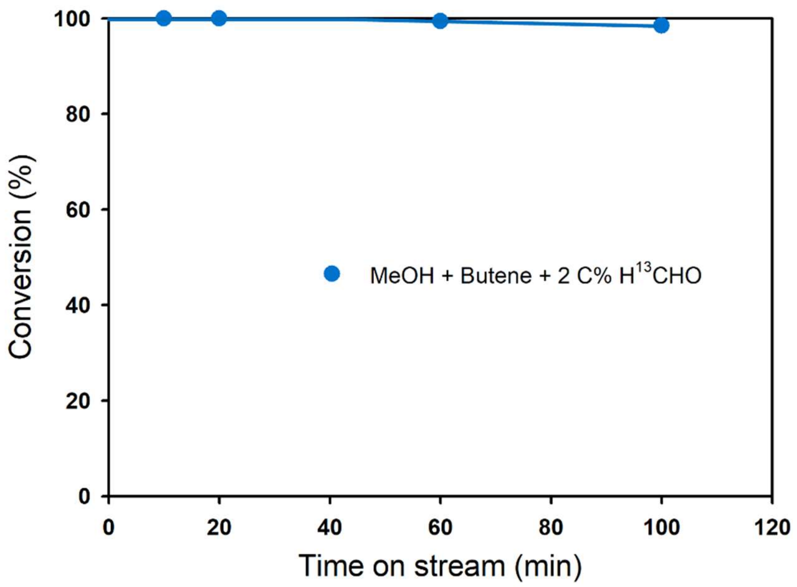

Supplementary Figure 2 | Evolution of MeOH conversion during MTO reaction with time on stream under the feeding of MeOH, 1-butene and 2 C% $H^{13}CHO$. Reaction conditions: W/F 0.96 $h \cdot g_{(cat)} \cdot mol_{(MeOH+HCHO)}^{-1}$, MeOH 171 mbar, $H^{13}CHO$ 9 mbar, H_2O 60 mbar, butene 60 mbar.
